# Field coupling as an interpretative framework for peptide–membrane interactions

**DOI:** 10.1007/s12551-026-01451-w

**Published:** 2026-08-18

**Authors:** Lúcio Otávio Nunes

**Affiliations:** Department of Systems Analysis and Development, Federal University of Jequitinhonha and Mucuri Valleys, MGT 367 Road, Km 583, N. 5000, Alto da Jacuba, Diamantina, Minas Gerais Brazil

**Keywords:** Antimicrobial peptides, Peptide–membrane interactions, Surface-bound state, Membrane insertion, Field coupling, Membrane biophysics

## Abstract

Antimicrobial peptides (AMPs) interact with lipid membranes through multistep processes that may include interfacial adsorption, partial insertion, membrane deformation, pore formation, and transmembrane organization. Although these processes have been extensively investigated, a unified operational framework connecting surface-bound, inserted, and intermediate peptide–membrane states remains underdeveloped. Here, we present a regime-based field-coupling framework that organizes peptide–membrane interactions according to the relative predominance of interfacial electrostatic coupling, hydrophobic-core coupling, and mixed coupling contributions. Surface-bound states are characterized primarily by interactions between cationic peptide residues and anionic lipid headgroups, whereas inserted states display stronger engagement of nonpolar peptide surfaces with lipid acyl chains. Mixed and pore-associated states retain both polar and hydrophobic interactions and may additionally involve water penetration, lipid-headgroup reorientation, membrane thinning, curvature, and peptide oligomerization. The operational use of the framework integrates peptide charge and charge distribution, hydrophobic moment, length and topology, membrane anionic charge, acyl-chain saturation, bilayer thickness, and peptide concentration. These variables are related to coordinated computational and experimental observables, including insertion depth, peptide–headgroup contacts, nonpolar residue–acyl-chain contacts, anionic lipid enrichment, peptide orientation, membrane order, water penetration, deformation, and peptide–peptide association. The framework generates conditional and falsifiable predictions for surface-bound, inserted/core, carpet-like, barrel-stave, and mixed/toroidal regimes. Its application to representative peptide–membrane systems illustrates how published observations can be organized within a common operational map. The proposed framework remains qualitative, and quantitative boundaries between coupling regimes require validation across peptides and membranes with distinct physicochemical properties. Nevertheless, it provides a structured basis for comparing mechanisms, designing computational and experimental tests, and interpreting transitions among peptide–membrane interaction states.

## Introduction

As antimicrobial peptides (AMPs) emerge as promising alternatives to conventional antibiotics, understanding their mechanisms of action becomes increasingly important. Because many AMPs exert their activity by targeting bacterial membranes (Karmakar et al. 2024; Oliveira Júnior et al. 2025), clarifying the physical principles that govern peptide–membrane interactions is particularly valuable. These interactions are complex and often involve multiple stages: some peptides remain predominantly surface-bound, others insert into the membrane core, and several follow sequential pathways in which initial surface association precedes partial or full insertion (Huang 2000; Nunes 2026). Although these processes are commonly described through energetic, structural, or molecular-interaction descriptors, additional conceptual perspectives may help clarify how different interaction regimes are connected within a common physical picture (Ma et al. 2024) .

The surface-bound stage of peptide–membrane interaction is a fundamental component of AMP action. This step primarily involves the interfacial region of the membrane, which separates the aqueous phase from the hydrophobic interior formed by phospholipid acyl chains (Lazzaro et al. 2020). The polar headgroup region may be zwitterionic or negatively charged, and the relative abundance of these lipid species determines the electrostatic character of the membrane surface (Yasir 2019). Electrostatic attraction therefore plays a major role in the initial binding step: most AMPs are cationic, whereas bacterial membranes typically possess a net negative surface potential due to their enrichment in anionic lipids such as phosphatidylglycerol (PG) and cardiolipin (CL) (Epand and Epand 2011). This interfacial association has often been analyzed in terms of binding free energy, electrostatic contributions, lipid recruitment, and membrane perturbation (Nunes 2026).

Peptide insertion is generally considered a subsequent stage of the interaction and is mechanistically distinct from the initial surface-bound phase (Gamage and Pan 2025). The membrane core is highly hydrophobic, which constrains the types of non-covalent interactions that can occur between the peptide and the bilayer interior (Nagle and Tristram-Nagle 2000). As a result, hydrophobic interactions, commonly described in terms of dispersion forces and the entropic drive associated with water exclusion, play a central role, and the peptide must contain sufficiently hydrophobic or amphipathic structural elements to associate with the apolar core (Seelig 2004). The transition from surface binding to insertion therefore involves not only peptide–lipid affinity, but also membrane deformation, peptide orientation, lipid packing, and the energetic cost of entering a low-dielectric environment (Sani and Separovic 2016).

Importantly, adsorption and insertion have not always been treated as independent processes. The adsorption–insertion coupling model and related theoretical approaches have described peptide binding, partial penetration, membrane perturbation, and pore formation as coupled events influenced by peptide concentration, penetration depth, membrane elasticity, lipid packing, and curvature stress (Zemel et al. 2005). In these models, interfacially adsorbed amphipathic peptides can perturb lipid-chain organization, modify membrane elastic properties, promote curvature stress, and facilitate structural transitions when a critical surface density is reached. Thus, surface association is not merely a passive preliminary step, but can actively reshape the membrane free-energy landscape and contribute to later insertion or pore-forming events (Zemel et al. 2004; Zemel et al. 2008).

Within this context, the present work proposes a field-oriented interpretative framework intended to reorganize established physicochemical interactions into a common conceptual language. The aim is to describe surface-bound and inserted regimes as related mechanisms, as distinct manifestations of peptide–membrane coupling occurring under different interfacial and intramembrane conditions.

In this framework, peptide–membrane interaction is viewed as a coupling between fields generated by the peptide and by the membrane. For clarity, we focus on the biologically relevant case of cationic peptides interacting with negatively charged membranes, as commonly observed for membrane-active antimicrobial peptides (Felsztyna et al. 2025). The initial surface-bound state arises from electrostatic attraction between cationic residues and anionic lipid headgroups. In the field-coupling picture, the peptide electrostatic potential interacts with the membrane surface potential to establish a stabilized interfacial configuration (Murray et al. 1999). Some peptides may subsequently insert into the membrane, a process that requires overcoming an energetic barrier associated with the disruption of this initial interfacial coupling (Irudayam et al. 2013). Once inserted, stabilization is no longer dominated by direct electrostatic coupling with headgroups, but by interactions within the hydrophobic core, commonly described as dispersion or induced-dipole interactions between hydrophobic peptide side chains and lipid acyl chains (de Planque and Killian 2003).

In this work, field coupling refers to the collective interaction between the spatially distributed electrostatic and electronic-density fields generated by peptides and membranes. The central message is that surface-bound association, insertion, and intermediate perturbation states can be interpreted within a unified framework in which the dominant form of coupling changes with peptide position, lipid environment, and membrane response. This conceptual perspective provides a common language for connecting interfacial electrostatic stabilization, adsorption–insertion coupling, and hydrophobic stabilization in the membrane core (Im and Brooks 2005). Figure 1 presents a conceptual representation of the proposed field-coupling regimes.

**Fig. 1:**
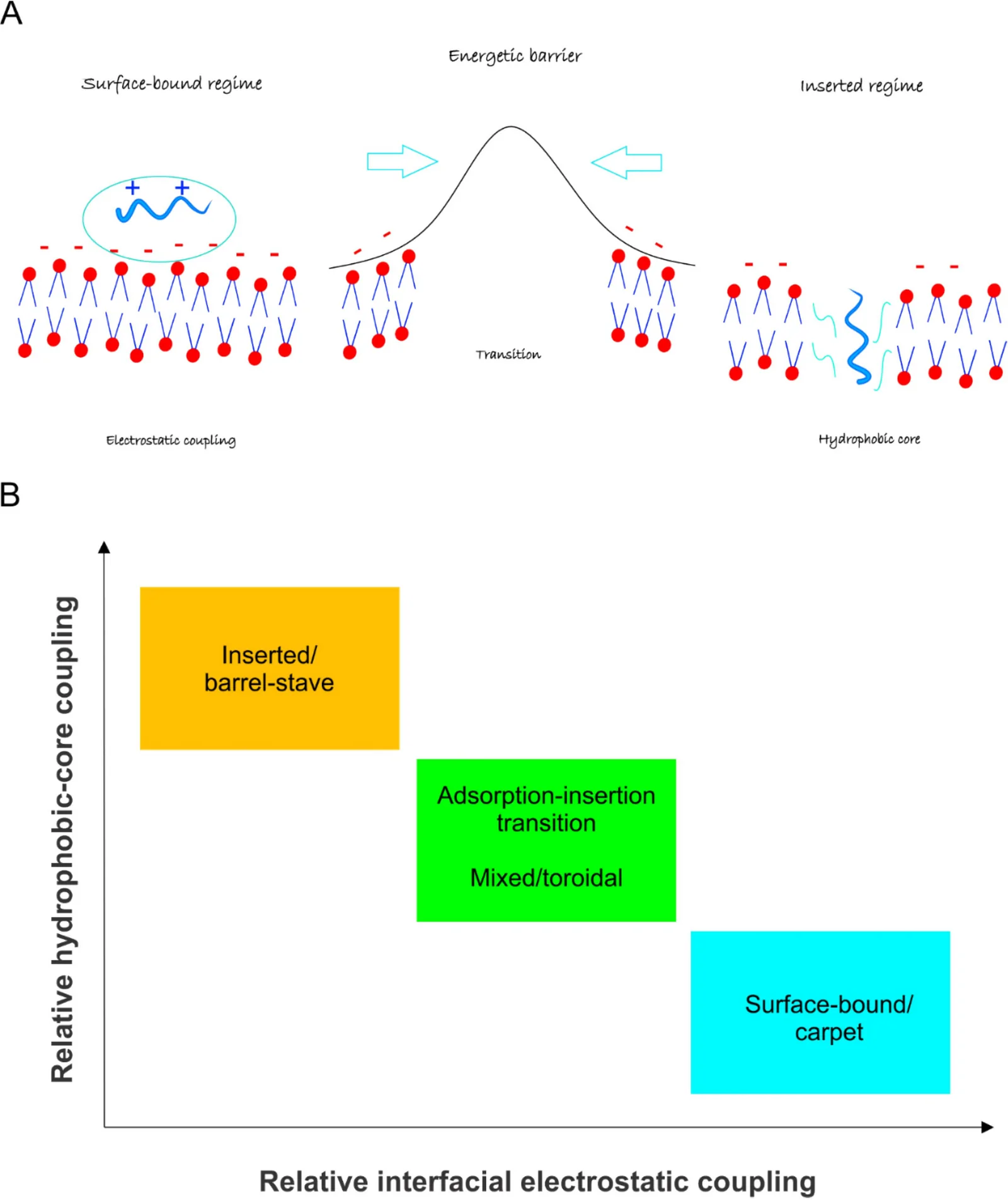
Conceptual representation (**A**) and operational mapping (**B**) of field-coupling regimes in peptide–membrane interactions

Figure 1A presents a representation of the transition from a surface-bound to an inserted peptide configuration. In the surface-bound regime, cationic peptides interact predominantly with anionic lipid headgroups through interfacial electrostatic coupling, which may promote local enrichment of anionic lipids and stabilize peptide association with the membrane interface. Progression toward insertion requires partial disruption or reorganization of this interfacial configuration and involves an energetic barrier associated with peptide reorientation, membrane deformation, desolvation, and penetration into the low-dielectric bilayer interior. Once insertion is achieved, peptide stabilization becomes increasingly associated with hydrophobic-core coupling, including nonpolar residue–acyl-chain contacts and dispersion- or polarizability-dependent contributions.

Figure 1B presents a qualitative regime map that positions representative peptide–membrane mechanisms according to the relative predominance of interfacial electrostatic coupling and hydrophobic-core coupling. Surface-bound and carpet-like states are associated with strong interfacial coupling and comparatively limited hydrophobic-core engagement. Inserted and barrel-stave-like configurations are associated with stronger hydrophobic-core coupling and reduced dependence on persistent interfacial electrostatic contacts. Adsorption–insertion transitions and mixed or toroidal states occupy an intermediate region in which electrostatic, hydrophobic, water-mediated, and membrane-elastic contributions coexist. The positions and boundaries shown in the map are conceptual rather than quantitative.

## Generalizable conceptual framework for membrane-active peptides

### Conceptual model: limiting and mixed field-coupling regimes

#### Surface-bound field coupling

A cationic peptide generates an electrostatic field through the spatial arrangement of its charged residues (Jiang et al. 2008). This field can couple to the electrostatic field produced by negatively charged membrane lipids. As the peptide approaches an anionic membrane surface, electrostatic interactions between cationic residues and anionic headgroups promote interfacial association, establishing a surface-bound field-coupling regime. In this framework, such coupling stabilizes the surface-bound state and contributes to the energetic and structural conditions that must be modified before deeper insertion can occur (Svirina and Terterov 2021).

Two possible pathways may then follow: the peptide may remain predominantly surface-bound, or it may progress toward partial or full insertion. In the first scenario, peptides accumulate at the membrane interface. Their combined peptide–membrane and peptide–peptide interactions can perturb the native electrostatic and mechanical landscape of the bilayer. Once a critical local peptide density is reached, the interfacial organization of the membrane may be disrupted, increasing configurational disorder and promoting membrane destabilization (Fernandez et al. 2012). This behavior is consistent with carpet-like mechanisms, in which membrane disruption arises primarily from peptide accumulation at the surface rather than from the formation of well-defined transmembrane pores (Shai 2002).

Insertion becomes more likely when peptide sequence, amphipathicity, hydrophobic moment, and membrane composition favor deeper penetration into the bilayer. To insert, the peptide must overcome the barrier associated with disrupting the initial surface-bound configuration and entering a low-dielectric, hydrophobic environment (Lyu et al. 2017).

#### Insertion and hydrophobic-core coupling

Some peptides transition from the surface-bound state to partial or full insertion into the membrane. This transition occurs when the interfacial configuration no longer fully stabilizes the peptide at the surface and when hydrophobic, amphipathic, and mechanical factors favor penetration into the bilayer interior (Schmidtchen et al. 2014). Within the field-coupling framework, insertion corresponds to the weakening or reorganization of the initial interfacial electrostatic coupling and the establishment of a different coupling regime in the low-dielectric environment of the membrane core (Takechi-Haraya et al. 2022).

Once inserted, the peptide interacts more extensively with the hydrophobic region of the bilayer. Hydrophobic side chains can associate with lipid acyl chains through interactions commonly described in physical chemistry as dispersion forces, hydrophobic effects, and induced-dipole interactions (Yin et al. 2012). Within the present framework, hydrophobic partitioning, solvent exclusion, dispersion interactions, and polarizability-dependent contributions are grouped operationally as hydrophobic-core coupling.

#### Intermediate states, water defects, and pore architectures

The two regimes described above should not be interpreted as an exhaustive classification of all peptide–membrane states. Rather, they represent limiting regimes within a broader continuum of membrane perturbation. In many AMP systems, especially those involving strongly amphipathic or hydrophilic peptides, membrane disruption may involve transient water defects, lipid headgroup intrusion into the bilayer interior, local thinning, curvature generation, and pore-like architectures. These configurations cannot be fully described by a simple distinction between surface-bound and inserted states (Khandelia et al. 2008).

Within the present framework, such structures are interpreted as mixed coupling states. In these states, interfacial electrostatic coupling is not completely lost, even when the peptide penetrates into the membrane. Instead, electrostatic interactions with lipid headgroups, water-mediated interactions, membrane deformation, and hydrophobic contacts with acyl chains may coexist. Toroidal pores, for example, may be viewed as hybrid configurations in which peptide insertion is accompanied by lipid bending and the participation of polar headgroups and water molecules within the membrane interior. Barrel-stave-like pores, by contrast, are expected to show a stronger contribution from hydrophobic-core coupling and peptide–peptide organization (Strömstedt et al. 2010).

The framework therefore provides a regime-based language for describing how interfacial, hydrophobic, water-mediated, and membrane-elastic contributions coexist during pore formation and other intermediate states.

### Relationship to classical AMP mechanisms

The proposed field-coupling framework is not intended to replace classical models of antimicrobial peptide action. Instead, it provides an interpretative layer through which these models can be compared in terms of the dominant coupling regime involved. Classical mechanisms such as the carpet model, barrel-stave pores, toroidal pores, and adsorption–insertion pathways can be understood as different structural outcomes arising from distinct balances among interfacial electrostatics, hydrophobic insertion, peptide–peptide association, membrane elasticity, lipid rearrangement, and water penetration (Brogden 2005; Bechinger and Gorr 2017).

In the carpet model, peptide action is dominated by surface-bound interactions. Cationic and amphipathic peptides accumulate at the membrane interface, often driven by electrostatic attraction to anionic lipid headgroups and by amphipathic partitioning at the polar–apolar boundary. At sufficiently high surface density, cooperative peptide accumulation can disrupt lipid packing and produce detergent-like membrane destabilization without requiring stable transmembrane pore formation. From the field-coupling perspective, the carpet mechanism corresponds mainly to an interfacial coupling regime, in which peptide–membrane interactions remain concentrated at the membrane surface and destabilization emerges from collective perturbation of the interfacial field and membrane organization (Talapko et al. 2022).

The barrel-stave model represents a more insertion-dominated mechanism. In this case, peptides insert into the bilayer and assemble into transmembrane bundles. The hydrophobic surfaces of the peptides interact with lipid acyl chains, whereas the polar faces line the aqueous pore lumen. Unlike toroidal pores, the lipid headgroups do not continuously bend through the pore to line the channel. From the field-coupling perspective, the barrel-stave mechanism corresponds to a regime in which hydrophobic-core coupling and peptide–peptide association become dominant, allowing a stable inserted architecture to form (Zhang et al. 2021).

The toroidal pore model represents a hybrid regime. Peptides insert into the membrane, but the pore is lined not only by peptide residues but also by lipid headgroups that bend continuously from one leaflet to the other. This mechanism therefore requires substantial membrane deformation, reorganization of lipid packing, and penetration of water and polar groups into the hydrophobic core. Hydrophilic or strongly amphipathic peptides may stabilize transient water defects or lipid-lined pores even when they do not behave as classical hydrophobic transmembrane helices. Within the field-coupling framework, toroidal pores correspond to a mixed coupling regime, in which interfacial electrostatic coupling, hydrophobic insertion, lipid-headgroup reorientation, water defects, and membrane curvature are simultaneously involved (Ludtke et al. 1996, Seyfi et al. 2020).

The adsorption–insertion model can be viewed as a transition pathway between predominantly surface-bound and inserted regimes. In this view, initial adsorption lowers the free energy of peptide association with the membrane surface, whereas increasing peptide surface density, membrane deformation, changes in lipid packing, and local curvature stress may facilitate penetration into the bilayer. The transition from adsorption to insertion is therefore not a simple switch between two independent states, but a coordinated reorganization of peptide position, peptide orientation, lipid environment, and membrane mechanical response (Huang 2000).

From the field-coupling perspective, these classical mechanisms differ not because they involve unrelated physical principles, but because different forms of coupling dominate in different structural regimes. Thus, the framework provides a common language for describing how electrostatic, hydrophobic, water-mediated, and membrane-elastic contributions are reorganized as peptides move from the membrane interface toward the bilayer interior.

## Operational use of the framework

The field-coupling framework can be operationally applied by integrating the dominant coupling regime with the physicochemical properties of both the peptide and the membrane. No single peptide or membrane property determines the interaction outcome. Rather, the relative predominance of coupling regimes emerges from the combined influence of peptide charge, charge distribution, hydrophobicity, amphipathicity, chain length, membrane surface charge, lipid packing, bilayer thickness, and peptide concentration. These parameters modulate the relative contributions of interfacial electrostatic coupling, hydrophobic-core coupling, and mixed coupling states. Accordingly, the framework provides a structured basis for relating system properties to measurable observables and for generating conditional predictions regarding whether a peptide is more likely to remain surface-bound, undergo partial or full insertion, or promote mixed and pore-like membrane perturbations. Each subsection therefore identifies both the expected influence of a system property on the balance among coupling regimes and the principal observables through which that influence can be evaluated.

### Peptide charge and charge distribution

Net positive charge, the spatial distribution of cationic residues, and the orientation of peptide or protein dipole moments are important determinants of membrane binding, interfacial orientation, and electrostatic coupling with the membrane surface potential (Galassi et al. 2018; Zheng et al. 2020). These properties define not only the magnitude of the peptide electrostatic potential but also its spatial organization and directionality.

The distribution of charge along the peptide sequence strongly influences the resulting field-coupling pattern. Peptides bearing highly localized clusters of cationic residues generate a different spatial electrostatic profile from peptides in which positive charges are more broadly distributed. When a peptide adopts an ordered conformation, such as an α-helix, the arrangement of charged residues and the orientation of the helix macrodipole may generate a more directional and spatially extended electrostatic potential. Consequently, both net charge and charge distribution can modulate the strength and geometry of peptide–membrane electrostatic coupling (Dathe and Wieprecht 1999).

From an operational perspective, increasing peptide cationic charge is expected, under otherwise comparable conditions, to strengthen interfacial electrostatic coupling with anionic lipid headgroups. This may increase peptide residence at the membrane surface, promote local recruitment of anionic lipids, and favor surface-bound or carpet-like configurations. However, high positive charge does not necessarily prevent insertion, because the final interaction regime also depends on hydrophobicity, amphipathicity, peptide conformation, membrane composition, and peptide concentration. Conversely, a lower net charge does not directly imply stronger induced-dipole or hydrophobic-core coupling; rather, it reduces the relative contribution of interfacial electrostatic stabilization, thereby allowing hydrophobic and insertion-related contributions to become comparatively more important (Dathe and Wieprecht 1999, Jiang et al. 2008; Yin et al. 2012).

The operational observables associated with peptide charge include the number and persistence of Lys/Arg–phosphate or Lys/Arg–anionic-headgroup contacts, peptide–membrane electrostatic interaction energy, local enrichment of anionic lipids, peptide residence time at the interface, and insertion depth.

### Hydrophobic moment and amphipathicity

Hydrophobicity and amphipathicity are closely related to the structural organization of antimicrobial peptides. Many AMPs adopt ordered secondary structures, particularly α-helices, that spatially segregate polar and nonpolar residues into distinct molecular faces. This organization allows the peptide to interact simultaneously with the membrane interfacial region through its polar or charged surface and with the apolar bilayer interior through its hydrophobic surface. The hydrophobic moment provides a quantitative descriptor of this directional segregation and therefore contributes to peptide orientation and partitioning at the membrane interface (Eisenberg et al. 1982).

Association between nonpolar peptide residues and lipid acyl chains is conventionally described in terms of hydrophobic partitioning, solvent exclusion, dispersion interactions, and polarizability-dependent contributions. Within the field-coupling framework, these effects are represented as spatially organized hydrophobic-core coupling between the nonpolar surface of the peptide and the hydrocarbon region of the bilayer. The magnitude and direction of the hydrophobic moment influence how this coupling is distributed over the peptide surface and may therefore affect whether the peptide adopts a surface-parallel orientation, penetrates partially into the bilayer, or forms a more deeply inserted configuration (Dathe and Wieprecht 1999).

From an operational perspective, a high hydrophobic moment is not, by itself, sufficient to predict membrane insertion. Strong amphipathicity may stabilize either an interfacial state, in which polar and nonpolar peptide faces remain aligned with the membrane–water boundary, or an inserted state, depending on net charge, overall hydrophobicity, peptide length, membrane packing, and bilayer thickness. Accordingly, the relevant prediction concerns the coordinated behavior of several observables rather than the hydrophobic moment in isolation (Dathe and Wieprecht 1999, Takechi-Haraya et al. 2022).

Observable signatures associated with hydrophobic moment and amphipathicity include peptide orientation relative to the membrane normal, insertion depth, the number and persistence of nonpolar residue–acyl-chain contacts, solvent-accessible surface area of hydrophobic residues, secondary-structure stability, and the simultaneous retention or loss of peptide–headgroup contacts.

### Chain length and topology

Peptide length and molecular topology influence field-coupling regimes by determining how charged and hydrophobic residues are distributed across the peptide structure and whether a sufficiently extended hydrophobic segment can interact with, or span, the membrane core. Under otherwise comparable conditions, a peptide whose hydrophobic length is compatible with the hydrophobic thickness of the bilayer may be more readily stabilized in a transmembrane configuration. Conversely, a mismatch between peptide length and bilayer thickness may favor peptide tilting, partial insertion, membrane deformation, oligomerization, or retention at the membrane interface (de Planque and Killian 2003).

Length alone, however, does not determine the interaction regime. A relatively long peptide may remain predominantly surface-bound if it has a high net charge, insufficient hydrophobic continuity, or a topology that favors interfacial orientation. Likewise, shorter peptides may penetrate the bilayer or promote pore-like states through cooperative association, membrane thinning, local defects, or lipid rearrangement. The effect of peptide length must therefore be interpreted together with hydrophobicity, amphipathicity, charge distribution, secondary-structure stability, membrane thickness, and peptide concentration (Khandelia et al. 2008, Sani and Separovic 2016).

Topology further modifies how these properties are spatially expressed. Linear, cyclic, helical, β-structured, or conformationally constrained peptides may display distinct orientations, flexibilities, aggregation propensities, and patterns of membrane contact. A topology that maintains a continuous hydrophobic surface may favor hydrophobic-core coupling, whereas a structure that exposes charged and polar residues toward the membrane interface may stabilize interfacial electrostatic coupling. Topologies that simultaneously preserve polar headgroup contacts and hydrophobic acyl-chain contacts may instead favor mixed or pore-associated coupling regimes (Schmidtchen et al. 2014; Ma et al. 2024).

Operational observables associated with peptide length and topology include insertion depth, peptide tilt relative to the membrane normal, hydrophobic mismatch, secondary-structure persistence, exposure of peptide termini, nonpolar residue–acyl-chain contacts, peptide–peptide association, and membrane thinning or curvature.

### Membrane anionic charge

Membrane anionic charge is a major determinant of the initial association between cationic peptides and lipid bilayers. An increase in the fraction of anionic lipids generally enhances the negative surface potential of the membrane and strengthens electrostatic attraction toward cationic peptide residues. Within the field-coupling framework, this effect increases the relative contribution of interfacial electrostatic coupling and may promote peptide recruitment, longer interfacial residence times, and local enrichment of anionic lipids around the bound peptide (Murray et al. 1999; Epand and Epand 2011; Svirina and Terterov 2021).

However, a highly anionic membrane does not necessarily prevent peptide insertion or pore formation. Strong electrostatic recruitment may stabilize a surface-bound configuration, but it may also increase the local peptide concentration at the membrane interface and thereby facilitate cooperative membrane perturbation, partial insertion, or pore formation. The resulting regime therefore depends on the combined effects of peptide hydrophobicity, amphipathicity, charge distribution, peptide concentration, lipid packing, and bilayer thickness. Membrane charge should consequently be interpreted as a variable that shifts the balance among coupling regimes rather than as an isolated determinant of peptide location (Huang 2000; Zemel et al. 2005; Melo et al. 2009).

Operational observables associated with membrane anionic charge include membrane surface potential, peptide-binding affinity, the number and persistence of Lys/Arg–phosphate or Lys/Arg–anionic-headgroup contacts, local anionic lipid enrichment, peptide surface density, insertion depth, and nonpolar residue–acyl-chain contacts.

### Lipid acyl-chain saturation and unsaturation

Lipid acyl-chain saturation influences peptide–membrane interactions by modulating bilayer packing, order, fluidity, thickness, and susceptibility to local deformation. Under comparable conditions, saturated acyl chains generally pack more efficiently and form more ordered membrane regions, whereas cis-unsaturated chains introduce conformational discontinuities that reduce packing efficiency and may increase membrane fluidity and the occurrence of transient packing defects. These differences can alter the energetic and structural conditions governing peptide adsorption, penetration, and membrane disruption (Pabst et al. 2008; Kumariya et al. 2015).

Within the field-coupling framework, lipid saturation does not directly determine the coupling regime. Rather, it modulates the accessibility of the hydrophobic core and the balance between interfacial electrostatic stabilization and deeper hydrophobic-core coupling. A more ordered and tightly packed bilayer may increase the barrier to peptide penetration and thereby favor prolonged interfacial residence, particularly for highly cationic or moderately hydrophobic peptides. Conversely, a more disordered bilayer containing unsaturated lipids may facilitate partial penetration, hydrophobic residue–acyl-chain contacts, membrane deformation, and the formation of transient water or headgroup defects. These effects are nevertheless conditional on peptide hydrophobicity, amphipathicity, charge distribution, bilayer phase state, acyl-chain length, and peptide concentration (Pabst et al. 2008; Beriashvili et al. 2018).

Operational observables associated with lipid saturation include acyl-chain order parameters, area per lipid, membrane thickness, lateral packing, peptide insertion depth, nonpolar residue–acyl-chain contacts, water penetration, lipid-headgroup intrusion, membrane curvature, and local thinning.

In pore-forming systems, increased peptide–peptide contacts may provide evidence of cooperative association or oligomerization, but they should be interpreted together with water continuity across the bilayer, headgroup reorientation, membrane thinning, and peptide insertion depth. The framework therefore predicts that changes in lipid saturation may shift the boundaries among surface-bound, inserted, and mixed coupling regimes rather than producing a universal effect on peptide activity.

### Bilayer thickness and hydrophobic mismatch

Bilayer thickness influences peptide–membrane interactions by determining the hydrophobic distance that a peptide must traverse or match to adopt a stable inserted configuration. Its effect cannot be interpreted solely in terms of thicker membranes hindering insertion and thinner membranes facilitating it. Rather, the relevant parameter is the degree of hydrophobic matching between the hydrophobic length of the peptide and the thickness of the membrane core. When these dimensions are compatible, an inserted peptide may be stabilized with relatively limited structural deformation. By contrast, hydrophobic mismatch may favor peptide tilting, partial insertion, oligomerization, local membrane thinning or thickening, or retention at the membrane interface (de Planque and Killian 2003).

Within the field-coupling framework, bilayer thickness modulates the energetic accessibility of hydrophobic-core coupling. For a peptide with a sufficiently long and continuous hydrophobic surface, a thicker membrane may support stable transmembrane insertion. The same membrane may, however, impose a larger insertion barrier on a shorter, highly charged, or only moderately hydrophobic peptide. Conversely, a thinner bilayer may facilitate penetration by some peptides, but may also generate an unfavorable mismatch for long transmembrane peptides. The resulting coupling regime therefore depends on the relationship among bilayer thickness, peptide hydrophobic length, peptide orientation, amphipathicity, and secondary-structure stability rather than on membrane thickness alone (de Planque and Killian 2003, Pabst et al. 2008).

Membrane curvature may further modify this relationship by generating local variations in lipid packing, leaflet area, and effective hydrophobic thickness. Curved or locally deformed regions may expose packing defects or produce membrane thinning, thereby shifting the balance between interfacial electrostatic coupling and hydrophobic-core penetration. However, curvature and thickness should be treated as related but distinct membrane descriptors, because curvature does not necessarily imply uniform thinning and its influence depends on leaflet asymmetry and the direction of bending (Zemel et al. 2008).

Operational observables associated with bilayer thickness include the distance between leaflet headgroup planes, local hydrophobic thickness, peptide insertion depth, peptide tilt relative to the membrane normal, hydrophobic mismatch, nonpolar residue–acyl-chain contacts, local thinning or thickening, and membrane curvature.

### Peptide concentration and cooperative effects

Peptide concentration is an important determinant of peptide–membrane behavior because it controls the local surface density of membrane-bound peptides and the probability of cooperative peptide–peptide and peptide–lipid interactions. At low peptide-to-lipid ratios, individual peptides may remain predominantly adsorbed at the membrane interface, particularly when interfacial electrostatic coupling provides the main source of stabilization. However, increasing peptide concentration does not necessarily lead directly to insertion. Instead, it may promote several concentration-dependent outcomes, including greater surface coverage, collective lipid-packing disruption, peptide oligomerization, partial insertion, pore formation, or detergent-like membrane disintegration (Melo et al. 2009, Tamba and Yamazaki 2009).

Within the field-coupling framework, increasing peptide concentration can shift the relative balance among coupling regimes by modifying the local electrostatic and mechanical environment of the membrane. Accumulation of cationic peptides at the interface may initially strengthen interfacial electrostatic coupling and increase local recruitment of anionic lipids. Once a critical surface density is reached, however, peptide crowding, membrane deformation, lipid redistribution, and peptide–peptide association may destabilize the surface-bound configuration and facilitate transitions toward inserted or mixed coupling states. The direction of this transition depends on peptide hydrophobicity, amphipathicity, topology, membrane charge, lipid packing, and bilayer thickness (Huang 2000, Zemel et al. 2005; Tamba and Yamazaki 2009).

Different mechanisms may therefore display distinct concentration-dependent signatures. In a carpet-like pathway, increasing peptide concentration is expected to produce high interfacial surface coverage, enhanced lipid disorder, and membrane destabilization without the formation of stable transmembrane structures. In barrel-stave-like systems, increasing concentration may favor peptide oligomerization and the formation of inserted transmembrane bundles. In toroidal or mixed pore-like states, higher local peptide density may promote simultaneous peptide insertion, lipid-headgroup reorientation, membrane curvature, and penetration of water into the bilayer core (Ludtke et al. 1996; Shai 2002; Qian et al. 2008; Tamba and Yamazaki 2009).

Operational observables associated with peptide concentration include the peptide-to-lipid ratio, local peptide surface density, peptide–peptide contact frequency, oligomer size, insertion depth, peptide–headgroup and peptide–acyl-chain contacts, lipid-order parameters, membrane thinning, water continuity across the bilayer, and membrane leakage.

### Operational assignment of coupling regimes

Operational regime assignment is based on the coordinated observables summarized in Table 1.

**Table 1:** Representative observables proposed here to distinguish peptide–membrane coupling regimes

Property	Surface-bound coupling	Inserted/core coupling	Mixed/toroidal coupling
Insertion depth into hydrophobic core	Low	High	Intermediate to high
Distance from bilayer midplane	Larger	Smaller	Variable
Lys/Arg–PO_4_ or headgroup contacts	High	Low to moderate	High or persistent
Local enrichment of anionic lipids	High	Lower or less decisive	Variable to high
Hydrophobic residue–acyl chain contacts	Low to moderate	High	Moderate to high
Peptide–peptide contacts	Low to moderate	High in oligomeric pores	Moderate to high
Water/headgroup penetration into the core	Low	Low to moderate	High
Membrane deformation/curvature	Mainly interfacial	Core-associated perturbation	Strong curvature and lipid rearrangement
Dependence on anionic lipid headgroups	Strong	Weaker after insertion	Strong during pore stabilization

The framework can be applied operationally to peptide–membrane systems by using the coordinated observables summarized in Table 1. The purpose of the following procedure is to determine the relative predominance of interfacial electrostatic coupling, hydrophobic-core coupling, and mixed coupling contributions.

First, observables associated with interfacial electrostatic coupling should be quantified. These include peptide–headgroup contacts, Lys/Arg–phosphate interactions, local enrichment of anionic lipids, peptide residence at the membrane interface, and peptide orientation relative to the bilayer surface.

Second, observables associated with hydrophobic-core coupling should be evaluated. Relevant measurements include peptide insertion depth, distance from the bilayer midplane, nonpolar residue–acyl-chain contacts, hydrophobic solvent exposure, peptide tilt, and hydrophobic matching between the peptide and the bilayer.

Third, signatures of mixed or pore-associated states should be identified. These may include the simultaneous persistence of polar and hydrophobic contacts, water or lipid-headgroup penetration into the membrane core, local thinning, membrane curvature, lipid rearrangement, and peptide–peptide association.

The resulting set of observables should then be compared with the coordinated patterns summarized in Table 1. A system may be assigned to a predominantly surface-bound, inserted/core, or mixed coupling regime according to the relative balance among these signatures. The same data can subsequently be used to position the peptide–membrane system within the qualitative regime map presented in Fig. 1B.

Because the framework is currently qualitative, regime assignment should be interpreted comparatively rather than as a strict threshold-based classification. Systems located near the boundaries of the map may display overlapping signatures or undergo transitions among regimes as peptide concentration, membrane composition, lipid packing, temperature, or other environmental conditions change.

## Testable predictions

The following predictions translate the qualitative regime map into coordinated signatures that can be tested computationally and, where appropriate, experimentally.

### Surface-bound regime

If interfacial electrostatic coupling is the dominant interaction regime, the peptide is expected to remain preferentially localized within the membrane headgroup region rather than penetrating deeply into the hydrophobic core. Molecular dynamics simulations should therefore reveal a coordinated signature consisting of a high frequency and persistence of Lys/Arg–phosphate or Lys/Arg–anionic-headgroup contacts, local enrichment of anionic lipids around the peptide, a relatively large distance between the peptide center of mass and the bilayer midplane, and comparatively few contacts between nonpolar peptide residues and lipid acyl chains.

Under otherwise comparable conditions, this signature should become more pronounced as peptide cationic charge or membrane negative surface potential increases, provided that peptide hydrophobicity and amphipathicity are insufficient to drive stable core penetration. Additional supporting observables may include a predominantly surface-parallel peptide orientation, prolonged interfacial residence, limited water or headgroup penetration into the membrane core, and the absence of persistent transmembrane peptide assemblies.

The prediction would be supported if these observables change coordinately toward stronger interfacial association. Conversely, it would be weakened if strong peptide–headgroup contacts were not accompanied by interfacial localization and reduced hydrophobic-core contacts, or if the peptide consistently adopted a deeply inserted configuration without the hybrid signatures expected for a mixed coupling state.

### Inserted/core regime

If hydrophobic-core coupling becomes the dominant interaction regime, the peptide is expected to penetrate more deeply into the bilayer and to establish sustained contacts between its nonpolar residues and lipid acyl chains. Molecular dynamics simulations should therefore reveal a coordinated signature consisting of increased insertion depth, a smaller distance between the peptide center of mass and the bilayer midplane, increased nonpolar residue–acyl-chain contacts, and reduced solvent exposure of hydrophobic peptide surfaces.

Relative to a predominantly surface-bound state, the inserted/core regime should also display a reduced contribution from persistent peptide–headgroup contacts and local anionic lipid recruitment. This does not imply that electrostatic interactions become irrelevant because anionic lipids may remain important for the initial adsorption, orientation, or cooperative insertion of the peptide. Rather, the prediction is that stabilization of the final inserted configuration becomes less dependent on interfacial electrostatic contacts and more strongly associated with interactions within the hydrocarbon region of the membrane. Stable secondary structure, favorable hydrophobic matching, and, in oligomeric states, increased peptide–peptide association would provide additional support for inserted/core coupling.

The prediction would be supported if deeper peptide penetration occurs together with increased acyl-chain contacts, reduced hydrophobic solvent exposure, and a relative decrease in interfacial electrostatic signatures. Conversely, it would be weakened if increased insertion depth were not accompanied by greater hydrophobic-core contacts, or if the peptide remained strongly dependent on headgroup interactions and water or lipid-headgroup penetration into the bilayer core. The latter pattern would be more consistent with a mixed or toroidal coupling regime than with a predominantly inserted/core regime.

### Mixed or toroidal coupling regime

If a mixed coupling regime develops, the peptide is expected to penetrate the bilayer while retaining substantial interactions with polar lipid headgroups and, in some cases, interfacial water. Molecular dynamics simulations should therefore reveal a hybrid and coordinated signature in which increased insertion depth and nonpolar residue–acyl-chain contacts occur without the complete loss of peptide–headgroup interactions.

Additional signatures may include water penetration into the hydrophobic core, lipid-headgroup intrusion toward the bilayer interior, local membrane thinning, curvature generation, lipid reorientation, intermittent water occupancy across the bilayer, and changes in leaflet packing. These observables need not occur with equal magnitude or simultaneously in every system. Rather, their combined occurrence would indicate that electrostatic, hydrophobic, water-mediated, and membrane-elastic contributions remain concurrently involved in stabilizing the perturbed membrane state. Persistent polar contacts within the membrane interior would distinguish this regime from a predominantly hydrophobic inserted/core state.

The prediction would be supported if deeper peptide penetration were accompanied by persistent headgroup contacts and by one or more structural signatures of membrane reorganization, such as water defects, headgroup displacement, thinning, curvature, or lipid rearrangement. Conversely, it would be weakened if peptide insertion occurred together with complete loss of interfacial polar contacts and without measurable membrane or water reorganization, a pattern more consistent with a predominantly inserted/core regime. It would also be weakened if the peptide remained exclusively surface-bound without significant penetration or intramembrane perturbation.

### Carpet-like mechanism

If peptide-mediated membrane disruption follows a carpet-like pathway, increasing the peptide-to-lipid ratio is expected to promote progressive peptide accumulation at the membrane interface, greater surface coverage, and collective perturbation of lipid organization without the formation of stable, well-defined transmembrane peptide assemblies. Molecular dynamics simulations should therefore reveal a concentration-dependent increase in interfacial peptide density, persistent peptide–headgroup contacts, lipid-packing disorder, and fluctuations in membrane thickness or local curvature, while most peptides remain predominantly parallel or oblique to the membrane surface. Limited penetration into the upper acyl-chain region may occur, but the absence of persistent transmembrane oligomers or stable peptide-lined pores remains the principal distinguishing feature.

The prediction would be supported if increasing peptide concentration produced coordinated increases in surface coverage, lipid disorder, and membrane leakage or destabilization without stable transmembrane insertion or persistent pore architecture. Conversely, it would be weakened if membrane permeabilization were consistently associated with deeply inserted peptide bundles, long-lived transmembrane oligomers, or continuous peptide-lined pores, which would be more compatible with barrel-stave-like or mixed pore-forming regimes.

## Computational and experimental validation strategy

### Computational and experimental observables

At present, the proposed framework remains theoretical. Nevertheless, it can be translated into a combined computational and experimental validation strategy designed to determine whether its predicted coupling signatures are observed in peptide–membrane systems. This section defines complementary observables through which the framework can be tested, refined, and potentially falsified.

A minimal validation design should include representative membrane-active peptides with distinct physicochemical profiles: (i) a predominantly cationic peptide expected to favor surface-bound interactions, (ii) a more hydrophobic or amphipathic peptide with a greater propensity for membrane insertion, and, when relevant, (iii) peptides capable of producing mixed or pore-like states. These peptides should be examined in bilayers with controlled anionic lipid content, acyl-chain saturation, packing order, and thickness. Different peptide-to-lipid ratios should also be considered to evaluate concentration-dependent and cooperative transitions. The central criterion is not the behavior of any single observable, but the coordinated change among structural, energetic, and membrane-remodeling descriptors.

First, peptide insertion depth and hydrophobic-core coupling can be evaluated computationally by measuring the position of the peptide center of mass relative to the bilayer midplane, peptide density profiles along the membrane normal, nonpolar residue–acyl-chain contacts, and the solvent exposure of hydrophobic residues. Experimental counterparts include solid-state NMR, oriented circular dichroism, and depth-dependent fluorescence quenching. Surface-bound peptides are expected to remain localized near the lipid-headgroup region, whereas insertion-prone peptides should display deeper penetration, greater acyl-chain contact, and an orientation compatible with partial or transmembrane insertion (Bürck et al. 2008; Strandberg et al. 2008; Orioni et al. 2009; Remington et al. 2022).

Second, interfacial electrostatic coupling can be assessed in molecular dynamics simulations by quantifying Lys/Arg–phosphate and Lys/Arg–anionic-headgroup contacts, electrostatic interaction energies, peptide residence at the interface, ion redistribution, and local enrichment of anionic lipids. Experimentally, isothermal titration calorimetry and membrane-binding isotherms can quantify binding affinity and thermodynamic contributions, whereas measurements of membrane surface potential or zeta potential can provide information on electrostatic recruitment and charge neutralization. These methods do not measure residue-specific contacts directly, but, when combined with structural or spectroscopic techniques, they can provide evidence for a predominantly interfacial electrostatic coupling regime (Abraham et al. 2005; Freire et al. 2011; Paterson et al. 2017).

Third, mixed, adsorption–insertion, and pore-associated regimes should be evaluated by examining whether increased peptide penetration occurs together with persistent polar or headgroup contacts. Computational observables include water continuity across the bilayer, lipid-headgroup intrusion into the hydrophobic core, peptide oligomerization, membrane thinning, curvature, and simultaneous polar and hydrophobic contacts. Experimentally, dye-leakage assays and membrane conductance measurements can detect permeabilization, whereas solid-state NMR, scattering methods, microscopy, or spectroscopic measurements may provide structural information on peptide orientation and membrane remodeling. Leakage or conductance alone cannot distinguish carpet-like, barrel-stave, and toroidal mechanisms; these measurements must therefore be interpreted together with structural evidence of peptide insertion, pore architecture, and lipid rearrangement (Ludtke et al. 1996; Qian et al. 2008; Tamba and Yamazaki 2009; Fernandez et al. 2012; Ulmschneider and Ulmschneider 2018).

Fourth, lipid composition and peptide-induced membrane remodeling can be analyzed computationally through lipid radial distribution functions, local lipid fractions, anionic lipid enrichment, leaflet-specific organization, acyl-chain order parameters, membrane thickness, and curvature. Lipid-specific fluorescence approaches may provide experimental evidence of lipid redistribution, whereas small-angle X-ray scattering and small-angle neutron scattering can be used to assess changes in membrane thickness, curvature, lamellar organization, and global bilayer structure. Because scattering measurements do not necessarily resolve local anionic lipid enrichment by themselves, contrast variation or complementary lipid-specific probes may be required (Pabst et al. 2008; Sharma and Qian 2019; Nielsen et al. 2021).

### Application of the framework to representative peptide–membrane systems

To illustrate the operational use of the field-coupling framework, three well-characterized peptide–membrane systems were classified according to coordinated observables reported in the literature (Table 2). The purpose of this comparison is not to reinterpret these peptides independently of their established mechanisms, but to demonstrate how published structural and functional observations can be organized in terms of the relative predominance of interfacial electrostatic, hydrophobic-core, and mixed coupling.

**Table 2:** Illustrative application of the field-coupling framework to representative peptide–membrane systems

Representative system	Key properties and published observations	Operational field-coupling assignment	Mechanistic classification
Aurein 1.2 in DMPC and DMPC/DMPG membranes	Neutron reflectometry and solid-state NMR indicated predominantly superficial peptide localization and relatively subtle changes in bilayer structure. Quartz crystal microbalance, dye-leakage, and atomic force microscopy measurements showed peptide accumulation at the membrane interface followed by bilayer disruption and lysis, without evidence of a stable transmembrane peptide pore (Fernandez et al. 2012)	Predominance of interfacial electrostatic and amphipathic coupling, characterized by enhanced interaction with anionic membranes, surface localization, limited stable hydrophobic-core penetration, and collective membrane disorder at increasing peptide surface density	Surface-bound/carpet-like regime
Alamethicin in phosphatidylcholine bilayers	Hydrophobic, predominantly helical peptaibol capable of transmembrane insertion and concentration-dependent oligomerization. Its nonpolar external surface is compatible with the lipid acyl-chain region, whereas its polar surface contributes to the aqueous pore lumen. X-ray diffraction reconstructed the electron-density profile of a transmembrane pore and demonstrated a barrel-stave architecture composed of eight alamethicin helices (Qian et al. 2008)	Predominance of hydrophobic-core coupling, indicated by stable transmembrane orientation, extensive peptide–acyl-chain contacts, strong peptide–peptide association, and limited participation of lipid headgroups in lining the pore	Inserted/barrel-stave regime
Magainin 2 in phosphatidylcholine/phosphatidylglycerol membranes	Cationic amphipathic α-helical peptide displaying concentration-dependent orientation and strong association with the lipid-headgroup region. At low peptide-to-lipid ratios, magainin helices were predominantly parallel to the membrane surface and associated with lipid headgroups. Above a critical peptide-to-lipid ratio, a substantial peptide fraction reoriented perpendicular to the membrane. Neutron in-plane scattering detected water-filled pores and supported a toroidal architecture in which lipid monolayers bend continuously and lipid headgroups remain associated with inserted peptides (Ludtke et al. 1996, Tamba and Yamazaki 2009)	Coexistence of interfacial electrostatic and hydrophobic-core coupling. Increased insertion occurs without loss of peptide–headgroup association and is accompanied by water penetration, lipid bending, bilayer thinning, and concentration-dependent reorientation	Adsorption–insertion transition and mixed/toroidal regime

These classifications generate experimentally and computationally testable expectations. Increasing the anionic lipid fraction should preferentially strengthen the interfacial signature of Aurein 1.2, whereas stable alamethicin pores should retain strong hydrophobic-core and peptide–peptide signatures. Magainin 2 should display a concentration-dependent shift from an interfacial state toward a mixed regime, accompanied by peptide reorientation, water penetration, membrane thinning, and lipid-headgroup rearrangement. The framework would be weakened if these coordinated signatures were not reproducible across comparable membrane conditions.

## Membrane saturation, packing, and biological implications

Membrane physicochemical properties are central determinants of peptide–membrane interactions and of the relative accessibility of different field-coupling regimes. Membrane anionic charge primarily modulates the initial electrostatic recruitment of cationic peptides, whereas acyl-chain saturation, lipid packing, bilayer thickness, and membrane fluidity influence the energetic and structural accessibility of the hydrophobic core. These properties should therefore be regarded as complementary rather than interchangeable determinants of peptide binding, insertion, and membrane disruption (Pabst et al. 2008; Epand and Epand 2011; Kumariya et al. 2015).

The biological relevance of these variables lies in the ability of cells to remodel membrane lipid composition and acyl-chain saturation in response to environmental stress. Changes in membrane order and fluidity may therefore alter peptide susceptibility even when membrane surface charge remains similar (Kumariya et al. 2015; Beriashvili et al. 2018). Within the field-coupling framework, this leads to a testable prediction: the same peptide may occupy different coupling regimes in membranes with comparable anionic charge but different levels of acyl-chain saturation and packing order. Such a transition should be reflected in coordinated changes in peptide insertion depth, peptide–headgroup and peptide–acyl-chain contacts, membrane-order parameters, local thinning, water penetration, and membrane leakage.

## Scope and limitations

Several limitations of the present framework should be explicitly acknowledged. First, the proposed classification and the regime map are qualitative. No quantitative thresholds currently define the boundaries among surface-bound, inserted/core, and mixed coupling regimes. Accordingly, the position of a peptide–membrane system in the map should be interpreted comparatively, on the basis of coordinated observables, rather than as a strict or absolute classification.

Second, field coupling is not measured here as an independent physical variable. Instead, the predominance of a given coupling regime is inferred operationally from measurable structural, energetic, and membrane-remodeling observables, including peptide insertion depth, peptide–headgroup contacts, nonpolar residue–acyl-chain contacts, anionic lipid enrichment, water or headgroup penetration, membrane deformation, and peptide–peptide association. The values of these observables may depend on the experimental technique, simulation protocol, force field, sampling time, membrane model, and normalization procedure. Future studies will therefore be required to establish standardized metrics and determine whether reproducible quantitative boundaries can be defined.

Third, the application presented in Table 2 is retrospective and illustrative rather than a prospective validation of the framework. The selected peptide–membrane systems were already associated with established mechanistic interpretations in the literature, and the framework was used to organize their published observables. A stronger validation will require prospective studies in which the coupling regime is predicted before experimental or computational characterization and subsequently tested against independent measurements.

The proposed regimes should also be regarded as limiting regions within a continuum rather than as mutually exclusive categories. Peptide–membrane systems may display overlapping signatures, occupy intermediate states, or transition among regimes as peptide concentration, lipid composition, temperature, ionic strength, membrane phase state, or other environmental conditions change. Consequently, the framework is better suited to describing the relative predominance and evolution of coupling signatures than to assigning every system to a single permanent mechanism.

The present formulation focuses primarily on cationic peptides interacting with anionic lipid membranes. This scope captures an important class of antimicrobial peptide systems but does not encompass the full diversity of membrane-active molecules or biological membranes. Native membranes may contain asymmetric leaflets, multiple lipid species, sterols, glycolipids, membrane proteins, lipopolysaccharides, peptidoglycan, and other components that influence peptide accessibility, membrane organization, and antimicrobial activity. These factors are simplified or absent from the current framework (Epand and Epand 2011, Felsztyna et al. 2025).

In addition, hydrophobic partitioning, solvent exclusion, dispersion, polarizability-dependent interactions, and membrane-elastic effects represent distinct physical and energetic contributions. Their grouping under the operational term “hydrophobic-core coupling” is intended to facilitate regime-level comparison and should not be interpreted as implying that they constitute a single energetic component or molecular mechanism (Seelig 2004; Sani and Separovic 2016).

Finally, the framework does not yet provide quantitative predictions of antimicrobial potency, selectivity, minimum inhibitory concentration, or de novo peptide sequence design. Its present contribution is to organize peptide and membrane properties into testable mechanistic expectations and to identify coordinated observables that may distinguish interaction regimes. Systematic validation across broader peptide libraries and membranes varying in charge, acyl-chain saturation, thickness, packing order, and phase state will be required to calibrate, refine, and potentially falsify the proposed framework.

## Conclusion

Antimicrobial peptides remain promising candidates for the development of novel anti-infective strategies. However, their rational design and optimization require a clearer understanding of how peptide sequence, membrane composition, interfacial binding, insertion, membrane deformation, cooperative effects, and pore formation are connected within a coherent biophysical description.

In this work, we present a field-coupling framework that organizes peptide–membrane interactions into surface-bound, inserted/core, and mixed coupling regimes. The framework integrates established electrostatic, hydrophobic, dispersion-related, water-mediated, and membrane-elastic contributions according to their relative predominance in different structural states. From this perspective, carpet-like disruption, barrel-stave pores, toroidal pores, and adsorption–insertion pathways can be compared as distinct outcomes of coordinated changes in interfacial electrostatic coupling, hydrophobic-core engagement, peptide–peptide association, and membrane reorganization.

The operational contribution of the framework is to connect peptide and membrane properties with measurable computational and experimental signatures. The framework therefore provides a structured basis for distinguishing interaction mechanisms and generating conditional, testable predictions. However, quantitative calibration and prospective validation across broader peptide and membrane sets remain necessary.

Despite these limitations, the framework offers a practical way to organize existing experimental and simulation data and to formulate comparative tests across peptide–membrane systems. Future studies combining molecular dynamics simulations with structural, thermodynamic, spectroscopic, scattering, and membrane-permeabilization measurements may determine whether the predicted coordinated signatures reproducibly distinguish surface-bound, inserted, and mixed states. Although developed in the context of antimicrobial peptides, this regime-based approach may also be useful for other membrane-active systems involving charged, amphipathic, insertion-prone, or pore-forming biomolecules.

## Data Availability

No datasets were generated or analysed during the current study.
